# Vertical Plate for Flail Chest Repair

**DOI:** 10.4103/jctt.jctt_10_19

**Published:** 2019-12-30

**Authors:** Marcel Tafen, Alexa Giammarino, Ceyda Bertram, Roman Petrov

**Affiliations:** Department of Surgery, Albany Medical College, Albany, New York,; Department of Thoracic Medicine and Surgery, Temple University Hospital, Philadelphia, PA, USA

**Keywords:** Flail chest, rib plating, thoracoplasty, vertical plate, vertical rib osteosynthesis

## Abstract

Operative treatment of rib fractures in the context of flail chest and respiratory failure is a well-established approach. In-line rib osteosynthesis with plates is the standard treatment sufficient to eliminate flail, achieve sufficient stability, and create chest rigidity to improve the respiratory cycle and maintain reduction. However, bridging large skeletal defects with missing portion of ribs is very challenging, particularly in the absence of suitable anchoring rib fragments. We describe an unusual use of vertical plate rib osteosynthesis in a patient with traumatic flail chest, exacerbated by a prior thoracoplasty and severe osteoporosis.

## Introduction

Rib fractures have historically been managed with pain control and respiratory therapy; however, operative treatment of rib fractures in the context of flail chest and respiratory failure is increasing.^[1]^ The now well-established approach involves in-line rib osteosynthesis with horizontal plates. To achieve this, there must be an adequate foundation for screw fixation and plate stabilization. Despite the advantages of locked plating, osteoporosis creates additional difficulties with high rates of screw pullout and unstable constructs, necessitating additional maneuvers.

With aging, individuals are likely to face multiple comorbidities that interfere with and negatively impact their bone density and strength. Chronic steroid use and diabetes are common issues that will affect the ability of tissues to heal. Large chest wall defects can result in chest wall instability and alter respiratory mechanics, all requiring surgeons to be creative in order to restore the chest wall integrity, preventing lung herniation while maintaining flexibility.

We describe a combined approach of the use of vertical plate rib osteosynthesis in addition to in-line rib osteosynthesis in a severely osteoporotic patient with flail chest, in the setting of prior thoracoplasty.

## Case Report

**Figure 1:**
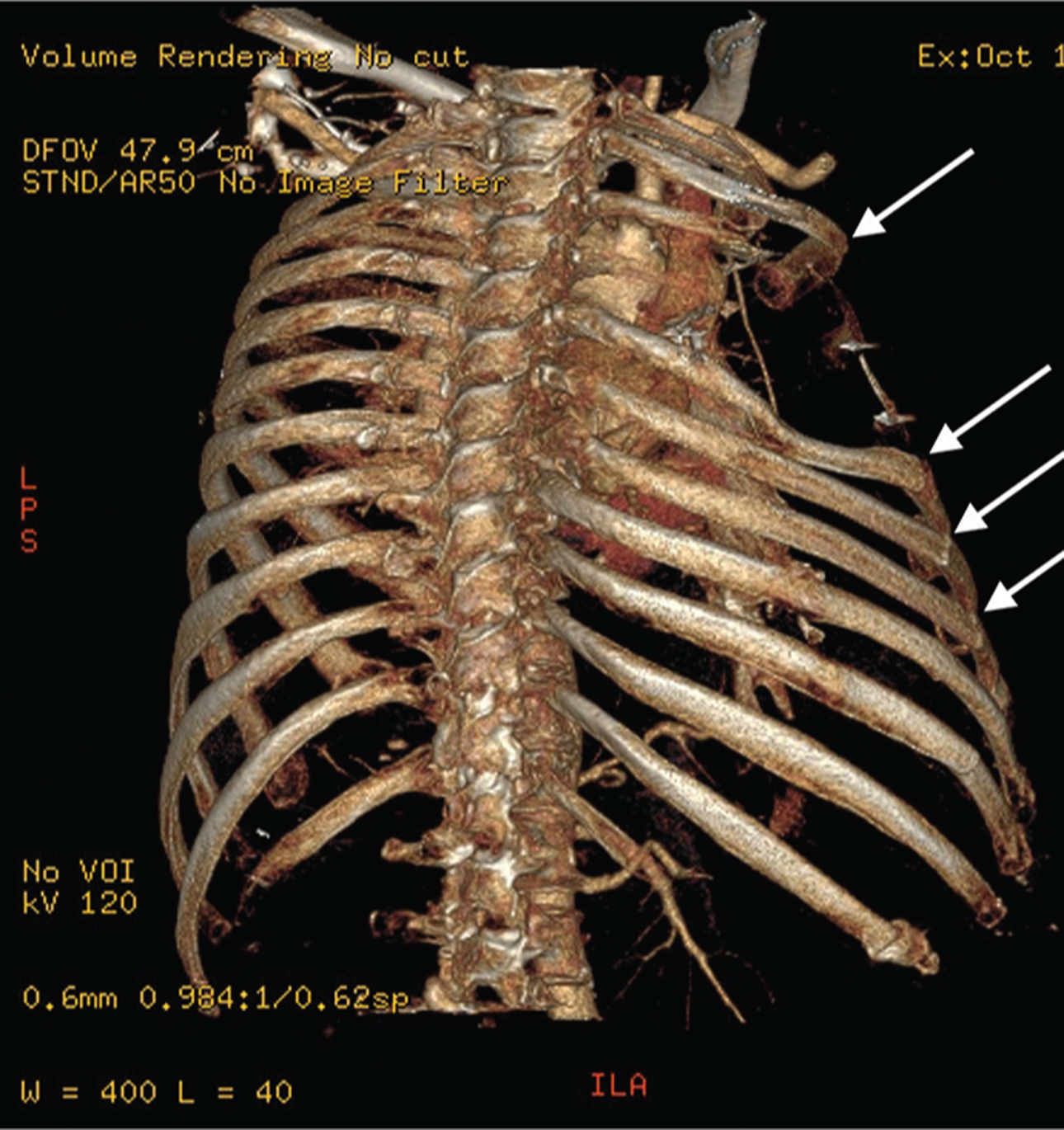
Three-dimensional volume rendering of computed tomography. Image displays the partial resections of ribs 3 and 4. Arrows indicate the lateral fractures of ribs 2, 5, 6, and 7

Mr. J.C. is a 60-year-old male with severe chronic obstructive pulmonary disease since 2005 requiring 4 L of oxygen at home and high-dose steroid therapy (prednisone 30 mg/day) for over 10 years, who was involved in a high-speed motor vehicle collision. He sustained a sternal fracture with mediastinal hematoma, multiple right rib fractures with flail chest, left acetabular fracture, left iliac fracture, and open proximal right tibial and fibular fractures [Figure 1]. His comorbidities are listed in Table 1.

The patient required high levels of oxygen on a high-flow nasal cannula and then was on bi-level positive airway pressure. Due to worsening respiratory status, the patient was intubated and taken to the operating room for surgical stabilization of flail chest 10 days after his admission.

**Figure 3:**
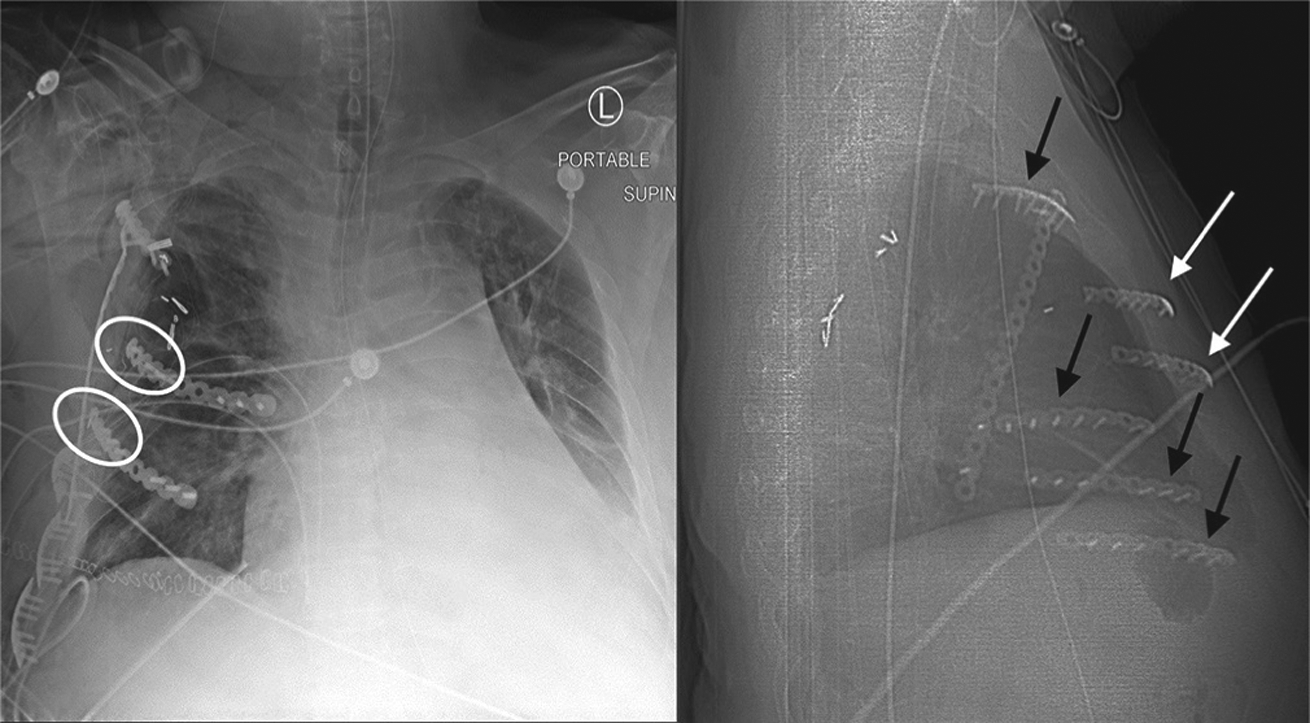
X-ray post repair. Horizontal plates spanning ribs 3 and 4 (white arrows), and ribs 2, 5, 6, and 7 (black arrows) are visualized. 1.0 Prolene was used to attach vertical plate to ribs 3 and 4 (circles)

A long thoracotomy incision was made using a muscle-sparing approach. Notably, the patient had prior right 3^rd^ and 4^th^ rib resections with posterolateral thoracoplasty after tumor resection in 1978. To compound this finding, the surrounding ribs 2, 5, 6, and 7 were fractured laterally. Residual segments of ribs 3 and 4 had comminuted fractures, anteriorly contributing to flailing. Severe atrophy of the cortex and osteoporosis was apparent, leading to immediate screw pullout during the case. Therefore, in-line bridging osteosynthesis of the long thoracoplasty segment was deemed to fail. The residual anterior 3^rd^ and 4^th^ rib segments were plated with an additional cerclage of the rib-plate construct using a Prolene suture (Ethicon, Somerville, NJ, USA). Further, in-line osteosynthesis of ribs 2, 5, 6, and 7 was accomplished, followed by a vertical rib plate using an 18-cm titanium plate anchored to the 2^nd^ rib superiorly and 5^th^ and 6^th^ ribs inferiorly. Prolene size 1.0 suture was used around the 3^rd^ and 4^th^ ribs to suspend the flailing thoracoplasty segment to the vertical plate [Figure 3]. The patient had immediate resolution of the flail. Chest tubes and subcutaneous drains were placed. The patient was extubated 48 h postoperatively and weaned to his home level of oxygen.

The patient had a prolonged hospital stay due to complexity of his polytrauma and burden of comorbidities. Interestingly, the patient developed recurrent left-sided atelectasis and effusions, requiring drainage, but no complications on the right chest.

## Discussion

Previous surgical repairs on patients with osteoporosis and flail chest secondary to multiple rib fractures have shown the need for increased measures when stabilizing the chest. In one patient with osteogenesis imperfecta, additional smaller 6-mm screws were added for plate fixation horizontally with additional reinforcement using Ethibond sutures (Ethicon, Somerville, NJ).^[2]^ The plating remained intact throughout the hospital course and follow-up. In another patient with osteoporosis secondary to cystic fibrosis, with multiple rib fractures, horizontal rib plating system and bone grafting were utilized.^[3]^ Berthet *et al*. described their experience in the use of 1–4 horizontal titanium bars in combination with DualMesh® (Gore, Flagstaff, AZ, USA), for repairing of multiple major chest wall defects.^[4]^ In a patient with a large posterolateral defect, fixation with a Vertical Expandable Prosthetic Titanium Rib (VEPTR) system (Synthes Spine Company, West Chester, PA, USA) for vertical osteosynthesis was performed. Although this device is mainly used on skeletally immature patients with constrictive chest wall syndrome or severely deformed rib cages, it was manipulated to successfully repair a large posterolateral full-thickness defect for the first time.^[4]^

This case illustrates the feasibility of a combined approach with vertical plate osteosynthesis in addition to in-line rib osteosynthesis. Moreover, vertical plating offers a viable alternative to the bridging of long flail segments with inadequate thoracic skeletal structure for in-line anchoring. To our knowledge, this is the first case of vertical rib osteosynthesis for flail chest in the literature.

## Figures and Tables

**Figure 2:**
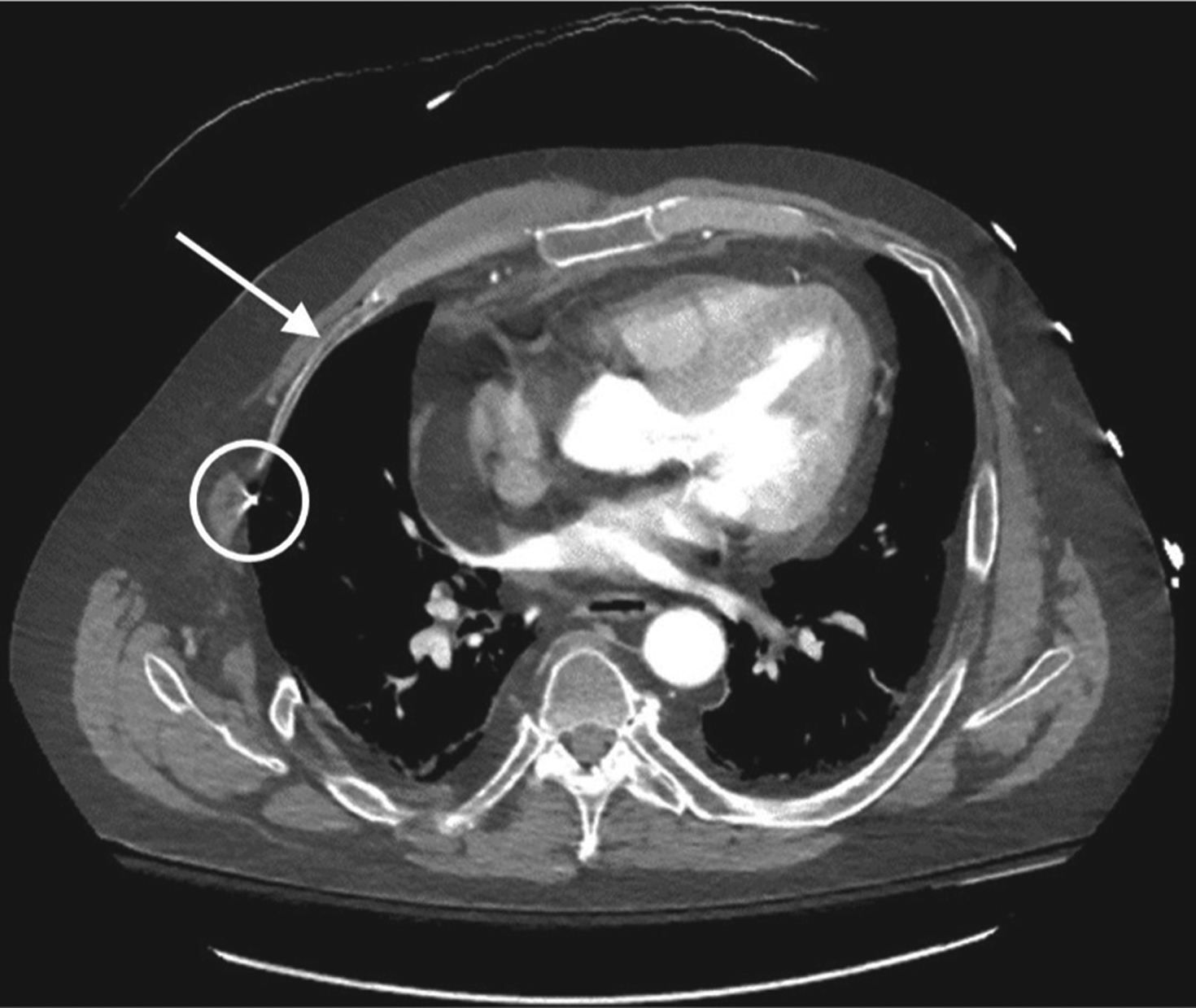
Computed tomography imaging of the chest showing clips (circle) from the patient’s previous right partial rib resection of ribs 3 and 4. Osteoporosis of the ribs is appreciated by the thinning of cortical bones (arrow)

**Table 1:** J.C.’s comorbidities

Partial removal of ribs 3 and 4 from tumor [Figure 2]
Branch duct intraductal papillary mucinous neoplasm of the pancreas
Metastatic prostate carcinoma
Severe COPD requiring home oxygen and steroids
Diabetes mellitus type 2
Bilateral hip fracture status post bilateral hip replacements
Asthma
Hypertension
Psoriasis
Pulmonary embolism
Hypercholesterolemia
Schwannoma
Cirrhosis
Hepatitis B
Class 1 obesity

COPD: Chronic obstructive pulmonary disease
